# Potential combination topical therapy of anal fissure: development, evaluation, and clinical study†

**DOI:** 10.1080/10717544.2018.1507059

**Published:** 2018-11-15

**Authors:** Amgad E. Salem, Elham A. Mohamed, Hosam M. Elghadban, Galal M. Abdelghani

**Affiliations:** a Faculty of Pharmacy, Department of Pharmaceutics, Mansoura University, Mansoura, Egypt;; b Department of Surgery, Mansoura University Hospital, Mansoura, Egypt

**Keywords:** Nifedipine, lidocaine hydrochloride, betamethasone valerate, anal fissure, combination topical therapy

## Abstract

To treat anal fissure, internal anal sphincterotomy may be associated with surgical risks and incidence of incontinence. Botulinum toxin injection into the anal sphincter is invasive and expensive. Headache and hypotension hindered topical treatment with glyceryl trinitrate. Greater patient compliance, potentiated efficacy, reduced side effects, and lower cost are the major advantages offered by the combination therapy. Therefore, combination topical gels of nifedipine (NIF), lidocaine hydrochloride (LDH) and betamethasone valerate (BMV) were prepared and evaluated regarding viscosity, pH, drug content, and *in vitro* release. Compatibility study of drug–drug and drug-excipient mixtures preceded the formulation. Stability study was performed. A prospective randomized clinical trial was conducted for six weeks to assess the efficacy of the optimized formula in the treatment of anal fissure either acute (AAF, 37 patients) or chronic (CAF, 34 patients) in comparison with three single drug market products. The compatibility was indicated except in case of LDH with each of poloxamer 407 (P407), methylparaben, and propylparaben as well as BMV with P407. The gels showed acceptable viscosity ranges, tolerated pH values, and drugs content limits complying with the pharmacopeial limit. The gel containing 10% Transcutol^®^ (F2) was selected as optimized formula due to the significant (*p* < 0.05) enhancement in NIF release. The recommended storage temperature was 8 °C. In comparison with the market products, the optimized gel can be represented as a potential combination therapy of acute and chronic anal fissures as suggested by significantly increased healing% and significantly reduced pain, bleeding, anal discharge and itching without side effects.

## Introduction

Anal fissure is one of the most painful anal disorders that inversely affect the life quality of the patients. It is an elliptical or longitudinal tear in the anoderm distal to the dentate line, exactly proximal to or at the level of the anal verge (Hall & Kann 2016). Anal fissures can be acute or chronic (Beaty & Shashidharan 2016). Acute anal fissure (AAF) is a simple rupture in the anoderm existing for less than two months and it is associated with anal pain, spasm, and/or bleeding with defecation. Chronic anal fissure (CAF) is present for more than 6–8 weeks and it is characterized by exposed fibers of internal anal sphincters at the base, hypertrophied anal papilla proximally, and a skin tag or sentinel pile distally.

Internal anal sphincterotomy has been used to heal the anal fissures through lowering the resting anal pressure. Yet, surgical risks and late permanent incidence of incontinence are the main complications during the post-operative period (Haq et al. 2005). Hence, alternative therapy regimens are necessary for the treatment of anal fissure. Chemical sphincterotomy has been induced by different agents including botulinum toxin, glyceryl trinitrate, and calcium channel blockers, such as diltiazem and nifedipine (NIF) (Perrotti et al. 2002). Use of topical nitrates is hindered by headache reported in as many as 90% of patients as well as orthostatic hypotension due to possible vasodilatation following systemic absorption (Bulus et al. 2013).

Calcium channel blockers (CCBs), such as NIF, cause smooth muscle relaxation and vasodilation resulting in increased blood flow and tissue irrigation that promote wound healing by this drug (Torsiello & Kopacki 2000). Factors that limit the use of topical nitrates are infrequent with CCBs making them an attractive alternative therapy. The use of either oral or topical NIF significantly improved the healing rates in patients with anal fissures providing comparable efficacy (Agrawal et al. 2013). Topical NIF at concentrations of 0.3 or 0.5% w/w effectively healed anal fissure (Perrotti et al. 2000). Local vasodilatation was induced by semi-solid formulations containing 8% w/w of NIF for wound healing without systemic effects (Torsiello & Kopacki 2000).

Fixed-dose combination drugs are formulations of two or more active ingredients in a single dose. Such combination is beneficial when the active ingredients are incorporated in the intended doses and in such way that the combination is safe and effective for a significant population of patients. Fixed combination therapy offers some advantages over concurrent administration of different single active ingredient preparations (Shenfield 1982). Among these improved patient compliance, enhanced efficacy, reduced side effects, and lower cost.

Fixed combination topical preparations containing a corticosteroid and a local anesthetic are extensively used to alleviate symptoms and improve the life quality of patients with inflammatory anal diseases. Corticosteroids act *via* binding to steroid receptors reducing inflammatory mediator’s synthesis, capillary dilatation, tissue infiltration, and intercellular edema, so symptomatic relief is not immediate (Havlickova 2010). Local anesthetics provide immediate relief of pain and itching after application. Therefore, an additive or synergistic effect in the fixed combinations of corticosteroids and local anesthetics is obtained (Havlickova 2010). Amide local anesthetics, such as lidocaine hydrochloride (LDH) are advantageous over ester-type since ester-type anesthetics that are metabolized to methyl-para benzoic acid causing allergic reactions and skin sensitization (Eggleston & Lush 1996). In accordance, the safety of repeated anorectal administration of 5% lidocaine ointment has been reported (Zimmermann et al. 2007). According to the Anatomical Therapeutic Chemical classification system developed by the World Health Organization, betamethasone is classified as a potent corticosteroid (Havlickova 2010). Subsequently, lidocaine has been commonly used in combination products with different corticosteroids including betamethasone (0.1% w/w) to treat the inflammatory anal diseases (Havlickova 2010). As well, combination products of NIF (0.3% w/w) and lidocaine (1.5% w/w) in form of ointment showed higher efficacy in treatment of anal fissure compared to a control topical ointment of lidocaine (1.5% w/w) and hydrocortisone acetate (1% w/w) (Perrotti et al. 2002).

In the light of the above-mentioned facts, it was worth to prepare, evaluate, and optimize a combination topical gel containing NIF, LDH, and betamethasone valerate (BMV). In addition, a prospective randomized clinical trial was performed to investigate the efficacy of the optimized combination formula in the treatment of AAF and CAF.

## Materials and methods

### Materials

Nifedipine (NIF) was kindly supplied by E.P.I.C.O pharmaceutical Co., Cairo, Egypt. Lidocaine hydrochloride (LDH), poloxamer 407 (P407), titanium dioxide, Tween^®^ 20 were purchased from Sigma-Aldrich, St. Louis, MO, USA. Betamethasone valerate (BMV) was kindly supplied by Pharaonic Company for Pharmaceutical Industries, Cairo, Egypt. Carbopol 940 (CP940) was purchased from BDH Chemical Ltd, Liverpool, England. Transcutol^®^ was obtained as a gift from Gattefosse, Saint-Priest, France. Methylparaben and propylparaben were obtained from El-Gomhoreyah Company for Trading Chemicals and Medical Appliances, Cairo, Egypt. Acetonitrile and methanol (HPLC grade) were purchased from Fisher Scientific Co., Loughborough, United Kingdom. Other chemicals were of fine analytical grade.

## HPLC analytical method

NIF, LDH, and BMV were quantified employing high performance liquid chromatography (HPLC) consisting of CMB-20 Alite system controller, LC-20 AD pump, DGU 20 A degasser system, and UV detector (Shimadzu, Japan), with LC Solutions version 1.3 software using a Promosil C_18_ column (250 × 4.6 mm, 5 μm, Agela Technologies, Wilmington, DE, USA). An assay based on a mobile phase of 146 µL trimethylamine and about 750 µL phosphoric acid added to 530 mL deionized water adjusted to pH of 3.3 using a 10% (w/v) KOH solution and 470 mL acetonitrile utilizing C_18_ column has been reported for quantification of the three drugs (Moffat et al. 2011). The mobile phase was filtered (0.2 µm) and degassed prior to use. Separation was carried out isocratically at a flow rate of 1.5 mL/min. Injection volume was 20 µL. UV detector was set at a wavelength of 240 nm. All assays were performed at ambient temperature.

The stock solution of NIF, LDH, and BMV was prepared at the same weight ratio (0.5:5:0.1, respectively) used in the prepared combination formulations. The stock solution was obtained by dissolving 100, 1000, 20 mg NIF, LDH, and BMV, respectively, in 100 mL methanol to give respective concentrations of 1000, 10,000, 200 µg/mL. Serial dilutions were made to obtain concentration ranges of 8–80, 80–800, and 1.6–16 µg/mL of NIF, LDH, and BMV, respectively. The standard calibration curve of each drug was constructed by plotting its concentrations in the prepared solutions versus the corresponding peak areas recorded.

## Drug–drug/drug-excipient compatibility studies

For the development of combination topical formulations of NIF, LDH, and BMV, differential scanning calorimetry (DSC), Fourier transform infrared spectroscopy (FT-IR), and isothermal stress testing (IST) were used to evaluate the drug–drug and drug-excipient compatibility (Verma & Garg 2005). The binary systems were prepared at a weight ratio of 1:1 to maximize the probability of detecting any interaction, if any, and minimize the dilution effect (Late & Banga 2008). The excipients examined were two gelling agents of carbopol 940 (CP940) and poloxamer 407 (P407), a penetration enhancer (Transcutol^®^), preservatives (methylparaben, propylparaben, and benzyl alcohol), and a photostabilizing agent (titanium dioxide). Transcutol^®^ has been used as a solubilizer and a penetration enhancer of NIF (Santis et al. 2013). Photodegradation of NIF has been reported (Maafi & Maafi 2013). Titanium dioxide has been employed as a photostabilizer (Yang et al. 2004).

### Differential scanning calorimetry (DSC)

Differential scanning calorimeter (Perkin-Elmer, model DSC-4, New York, NY) was used to study the thermal characteristics of drugs and 1:1 w/w physical mixtures of drug–drug and drug-excipients. Samples (4 mg) were heated in aluminum crimped pans under nitrogen gas flow at heating rates of 5 °C/min over a temperature range of 35–400 °C. Temperature calibration was performed using indium (99.99% purity, m.p. 156.6 °C) as a standard in DSC runs.

### Fourier transform infrared spectroscopy (FT-IR)

FT-IR spectra of drugs, excipients, drug–drug and drug-excipient 1:1 physical mixtures were recorded using FT-IR spectrophotometer (Thermo Fisher Scientific, Inc., Waltham, MA, USA). Disc of each sample with potassium bromide was individually scanned over a wavenumber range of 500–4000 cm^−1^.

### Isothermal stress testing (IST)

Drugs and different excipients were weighed and mixed to be placed in 10 mL glass vials (*n* = 3). A volume of water equivalent to 10% (w/w) of the sample was added and mixed with a glass rod. To prevent any loss of material, the glass rod was broken and left inside the vial. Each vial was sealed using a screw cap and stored at 50 °C (Hot air oven, Heraeus GS, model B 5042, Hanau, Germany) for four weeks. The controls were mixtures without added water and stored in refrigerator.

To detect the physical instability, organoleptic parameters of samples, such as color and odor, were observed at the end of the test. To identify the chemical instability, each sample was divided into two parts at the end of the fourth week. To the first part, a volume of 50 mL methanol was added to be vortexed, sonicated, centrifuged, filtered through 0.2 µm Millipore filter, and suitably diluted. Samples were analyzed quantitatively using the previously described HPLC method in triplicate. The second part was used to record FT-IR spectrum.

## Preparation of combination gel formulations

### Photostability study of NIF

ICH guideline Q1B for photostability testing has been reported (Aman & Thoma 2003). To determine the required concentration of titanium dioxide as a photostabilizer, this excipient was added to NIF gel prepared as illustrated below at different concentrations (1, 2, 3, and 4% w/w) and compared with NIF gel without the photostabilizer. Two grams of each formulation were irradiated in glass dishes with 5 cm diameter (Aman & Thoma 2003). The gel was carefully spread to give a layer as homogenous as possible with a suitable thickness. Samples were placed in direct sunlight between 12:00 noon and 2:00 pm in July 2017 at Mansoura, Egypt at a temperature ranged from 29 to 35 °C (Lodén et al. 2005). After irradiation, samples were analyzed to assess NIF content employing the previously illustrated HPLC method.

### Preparation of gel formulations

Based on the results of DSC, FT-IR, IST, and photostability studies as discussed later, two selected combination gel formulations, namely F1 (without Transcutol^®^) and F2 (with 10% w/w Transcutol^®^), were prepared. As a preliminary step, Transcutol^®^ was tried at different concentrations (4, 6, 8, 10, and 20% w/w) in the combination gel formulation and the maximum flux of NIF was obtained in case of 10% w/w Transcutol^®^, being insignificantly different from that observed with gel containing 20% w/w Transcutol^®^. The first gel formulation (F1) was prepared by dispersing CP940 (1.5% w/w) in double distilled water with a constant stirring and the resultant dispersion was kept overnight at a refrigerator. NIF (0.5% w/w) and BMV (0.1% w/w) were dispersed in double distilled water and then added to the gel base with continuous stirring until homogenous distribution. LDH (5% w/w) was dissolved in double distilled water and added with a proper mixing. Afterwards, titanium dioxide at the optimized concentration was dispersed in Tween^®^ 20 (2% w/w) and mixed with the gel. Benzyl alcohol at a concentration of 3% w/w was added and mixed well. The final weight of the gel was adjusted to 100 g. The pH was adjusted to 5.5–6 using triethanolamine to allow carbopol gelation (Khunt et al. 2012) and hinder isomerization of BMV since this compound showed a maximum stability at pH range of 4–6 (Byrne et al. 2017). The second gel formulation (F2) was prepared using the same procedure except that NIF and BMV were dissolved in Transcutol^®^ (10% w/w) and then added to the gel base with continuous stirring.

## *In vitro* evaluation of the studied formulations

### Drug content, pH, and viscosity

The evaluated formulations were the two combination gel formulations (F1 & F2) as well as Lignocaine^®^ 5% gel that contains 5% LDH (The Nile Co. for Pharmaceutical and Chemical Industries, Cairo, Egypt) and Betaderm^®^ cream with 0.1% BMV (E.I.P.I.CO., 10th of Ramadan, Egypt) as two marketing products of LDH and BMV, respectively, for comparison. One gram of each gel was vortexed with 25 mL methanol for 5 min and then sonicated for 15 min (ABBOTA Corporation, Princeton, NJ, USA) to extract the drugs. The mixture was centrifuged for 15 min. The supernatant was filtered through 0.2 µm Millipore filter and suitably diluted to be assayed utilizing the HPLC assay method previously illustrated. PH measurement was accomplished using a calibrated digital pH meter (Beckman Instruments Fullerton, CA 92634, Krefeld, Germany) in triplicate and the average values were calculated. The viscosity was estimated using a calibrated cone and plate rotary viscometer (Haake Inc., Vreden, Germany). The temperature was maintained at 37 ± 0.5 °C. One gram of each formulation was placed on the plate and the torque value (S) was determined to maintain the speed (N) at 256 rpm. Viscosity was calculated using the equation (*η* = G.S/N), where *η*:viscosity in (mPa.s), G:instrumental factor (14,200 mPa.s/scale grad. min), S: torque (scale grad.) and N: speed (rpm).

### *In vitro* release studies

*In vitro* release of NIF, LDH, and BMV from the formulations was studied using a specially designed diffusion cell (Arafa & Ayoub 2017). Cellophane membrane (molecular weight cutoff 14,000 Da., Fischer Sci. Co., Pittsburgh, PA, USA) was soaked before the experiment in the release medium of phosphate buffer pH 7.4 with 0.5% (w/v) sodium lauryl sulfate. The membrane was stretched over the open end of 3 cm diameter glass tube and was made watertight by a rubber band. Two grams of each formulation were equally spread on the cellophane membrane. The donor cells were immersed in 250 mL beaker containing 100 mL release medium maintained at 37 ± 0.5 °C in a thermostatically controlled shaking water bath (Grant Instrument Cambridge Ltd., Barrington Cambridge, B2, 5002, Royston, England). The donor cells height was adjusted so that the membrane was just below the release medium surface. The cells were shaken at 25 strokes per min. One-milliliter samples were withdrawn from the receiver compartment at 0.25, 0.5, 1, 2, 3, 4, 5, 6, 7, and 8 h and replaced by an equal volume of fresh medium at the same temperature.

The amounts of drugs permeated per 1 cm^2^ were determined using the previously illustrated HPLC method to be plotted against time. The intercept and slope of the linear portion of the plots were derived by linear regression. The steady-state permeation flux (*J*_ss_) for each drug in each formula was calculated as the slope divided by the membrane surface area (Das et al. 2013).

$$\text{Jss}=(\frac{dQ}{dt})ss\frac{1}{A}$$

Where *J*_ss_ is the permeation flux at steady-state (µg/cm^2^/h), A is the area of cellophane membrane (cm^2^), and (dQ/dt)_ss_ is the amount of drug permeated at steady state per unit time (µg/h).

### Release kinetics

In order to determine the release mechanism of drug, *in vitro* release data of each drug from the studied formulations were analyzed according to zero-order, first-order (Martin et al. 1993), and diffusion-controlled release mechanism (Higuchi 1963). The Korsmeyer–Peppas kinetic model was also employed (Ritger & Peppas 1987).

### Stability study

The optimized formulation was subjected to accelerated stability study according to the International Conference on Harmonization (ICH) guidelines for 3 months (Srivastava et al. 2005). Fifty grams of the formulation were placed in airtight collapsed aluminum tubes then, stored at different temperatures; 8 ± 1, 25 ± 1 and 40 ± 1 °C for three months. At time intervals of 0, 15, 30, 45, 60, 75, and 90 d, samples were examined for drugs content, pH, and viscosity.

### Shelf life analysis

Percentage remaining of each drug in the tested optimized combination gel was plotted against time in days to determine its shelf life (*t*_90_) and half-life (*t*_50_) at each storage temperature using *Sigma Plot 12* software (Cranes Software International, Bangalore, India) (Hooda et al. 2012; Nagajyothi et al. 2015).

### Microbiological stability

Sterility testing was done for aerobic bacteria using nutrient broth medium, anaerobic bacteria employing fluid thioglycollate medium at 37 ± 1 °C for 7 d, and fungi utilizing fluid sabouraud medium at 25 ± 1 °C for 10 d in incubator (Denyer & Hodges 2004). Sterility testing of the optimized combination gel was done after storage period (three months) at different temperature 8 ± 1, 25 ± 1 and 40 ± 1 °C. A quantity of 500 mg was taken from each sample using a sterile swab to be diluted in 100 mL sterile water, then 1 mL was added to 9 mL of each medium to be incubated for the specified time periods.

## Clinical study

### The study aim

The study was performed to evaluate the efficacy of the optimized topical combination gel of the investigated drugs in the treatment of acute and chronic anal fissures in comparison with three single drug topical market products. The market products were GTN cream that contains 0.2% w/w glyceryl trinitrate (A.U.G, Giza, Egypt), Lignocaine^®^ 5% gel of LDH (The Nile Co. for Pharmaceutical and Chemical Industries, Cairo, Egypt) and Betaderm^®^ cream with 0.1% BMV (E.I.P.I.CO., 10th of Ramadan, Egypt).

Research Ethics Committee of Faculty of Pharmacy, Mansoura University, Egypt approved the study and the included informed consents of all patients. The patients were allocated into four groups: group A; with acute anal fissure (AAF) treated with the optimized topical combination gel (F2), group B; with AAF treated with the three single drug topical market preparations within 0.5 h interval, group C; with CAF treated with the optimized formula (F2), group D; with CAF treated with the three topical market preparations within 0.5 h interval.

***Inclusion criteria:*** Patients who were aged between ≥18 and ≤65 years with either AAF or CAF**.**

***Exclusion criteria were:*** Presumed or confirmed pregnancy; lactating women; any history of reaction to topical agents and associated co-morbidity, such as ischemic heart disease, hypertension, diabetes mellitus, inflammatory bowel disease, HIV-related fissure, tuberculosis ulcer and leukemic ulcer; and associated complications warranting surgery (abscess, fistula, hemorrhoids, and cancer) as well as unwillingness of the patient to participate in the study (Tsunoda et al. 2012).

All enrolled patients were instructed to squeeze 1 cm of the gel onto a finger and to apply it inside the anus and to the anal margin. Each patient had given a 6-week course of the tested topical preparation for twice-daily application, as close to every 12 h as possible.

### Assessment

***Fissure healing:*** Objective changes were assessed by anus inspection to determine the extent of fissure healing (recorded as ‘healed’, ‘partially healed’, or ‘persistent’) at baseline (zero time) and weeks 1, 3, and 6. Anoscopy was performed at baseline and during the sixth week. Healing of anal fissure was defined at anoscopy when epithelialization or formation of a scar achieved at the sixth week of the therapy.

***Pain:*** The pain intensity was assessed according to a visual analog score (VAS) (Collins et al. 1997).

***Bleeding response, discharge, and pruritus:*** Patient scored the severity of their symptoms at baseline and weeks 1, 3, and 6, on numeral rating scales (NRS) (range: 0–10) (Tsunoda et al. 2012).

***Adverse effects of the medicines:*** such as itching, headache, and dizziness were recorded at every visit.

***The end-point of the study:*** it was complete healing of the fissure after treatment. Treatment was considered successful if the fissure had healed within 6-week treatment.

## Statistical analysis

With respect to the data of compatibility, drug release, and stability, statistical analysis was done through ANOVA (one-way analysis of variance) followed by Tukey–Kramer multiple comparisons test employing Graph Pad Prism-6 software (Graph Pad Software Inc., San Diego, CA, USA). Regarding the clinical study, the collected data were coded, processed, and analyzed. Predictive analytics software (PASW) version 22 (SPSS Inc., Chicago, IL) was used. Categorical data were represented as absolute numbers (frequency), while continuous data were expressed as mean values ± SD or median (inter-quartile range [IQR]), depending on if the data were normally distributed or not, as detected by the Kolmogorov–Smirnov Z test. Mann–Whitney U test was used to compare the continuous data. Chi-square test was used to compare the categorical data of the studied groups. *p* Value less than 0.05 was considered as statistically significant.

## Results and discussion

## HPLC analytical method

The peaks of NIF, LDH, and BMV were well-resolved at respective retention times of 5.8, 3.2, and 10.6 min. An excellent linearity was observed in a concentration range of 8–80, 80–800, and 1.6–16 µg/mL for NIF (*r*^2^ = 0.9992), LDH (*r*^2^ = 0.9987), and BMV (*r*^2^ = 0.9997), respectively. The equations describing the corresponding calibration curves were *y* = 39.70x + 19.87, *y* = 4.46x + 43.50, and *y* = 23.39x + 0.85767 where *y* represents the peak area of the drug and x represents the corresponding drug concentration (µg/mL).

## Drug–drug/drug-excipient compatibility studies

### Differential scanning calorimetry (DSC)

**Figure 1.**
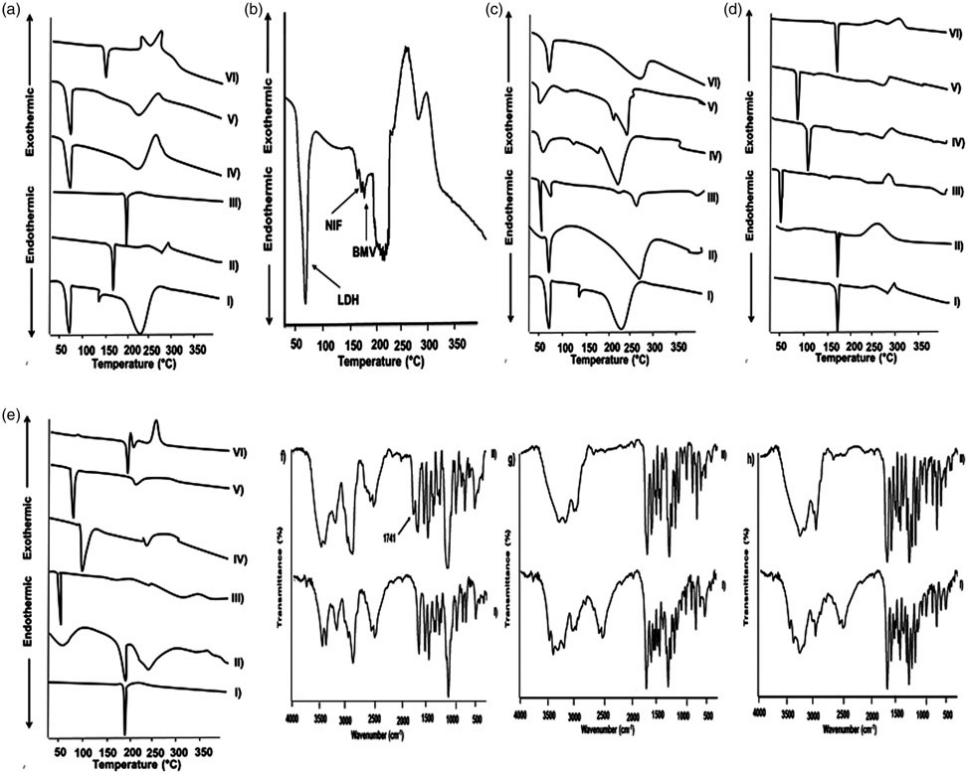
(a–e) DSC thermograms of LDH, NIF and BMV as (a) drug–drug mixtures where (I) LDH (II) NIF (III) BMV (IV) LDH-NIF (V) LDH-BMV (VI) NIF-BMV (b) tertiary drug mixture, (c) LDH-excipient mixtures, (d) NIF-excipient mixtures, and (e) BMV-excipient mixtures; where I) drug alone, II) drug-CP940, III) drug-P407, IV) drug-methylparaben, V) drug-propylparaben, and VI) drug-titanium-dioxide. (f–h) FT-IR spectra of (f) LDH-P407, (g) LDH-methylparaben, (h) LDH-propylparaben, as control sample (I) and sample subjected to isothermal stress (II).

DSC thermograms of the three drugs individually, in their binary mixtures and ternary system as well as binary mixtures with each of the solid excipients are represented in Figure 1(a–e). Peak temperature (*T*_peak_) values of drugs, drug–drug, and drug-excipient mixtures (1:1 w/w) are summarized in Table 1(a).

**Table 1.** (a) Peak temperature (*T*_peak_) values of drugs, drug–drug and drug-excipient mixtures (1:1 w/w). (b) IST results of drugs, drug–drug, and drug-excipient mixtures (1:1 w/w) after 4 weeks of storage at stressed conditions.

(a)						
Sample	*T*_peak_ (°C)					
	NIF		LDH		BMV	
Drug or drug–drug	–		–		–	
NIF	169.94		–		–	
LDH	–		77.15		–	
BMV	–		–		194.5	
NIF + LDH	Disappeared		73.49		–	
NIF + BMV	150.8		–		Disappeared	
LDH + BMV	–		75.3		Disappeared	
NIF + LDH + BMV	167.2		73.8		180	
Drug-excipient						
CP940	171.33		77.16		190.54	
P407	Disappeared		75.788		Disappeared	
Methylparaben	Disappeared		50		Disappeared	
Propylparaben	Disappeared		50.95		Disappeared	
Titanium dioxide	171.93		73.117		192	
(b)						

	Drug remained (%), mean ± SD					
	NIF		LDH		BMV	
Sample	Control	Test	Control	Test	Control	Test

Drug or drug-drug	–	–	–	–	–	–
NIF	100.00 ± 1.00	103.23 ± 0.76	–	–	–	–
LDH	–	–	99.39 ± 1.82	101.19 ± 1.10	–	–
BMV	–	–	–	–	99.80 ± 0.75	97.90 ± 0.50
NIF + LDH	102.00 ± 0.20	101.40 ± 2.50	102.60 ± 2.00	102.10 ± 7.70	–	–
NIF + BMV	103.00 ± 0.10	103.70 ± 1.00	–	–	103.20 ± 0.90	98.14 ± 1.00
LDH + BMV	–	–	101.36 ± 2.57	102.40 ± 0.60	102.10 ± 7.70	99.20 ± 0.50
Drug-excipient						
CP940	103.60 ± 1.22	101.00 ± 1.35	104.30 ± 1.57	104.38 ± 0.90	101.00 ± 0.80	97.00 ± 0.50
P407	104.00 ± 1.60	98.50 ± 1.50	104.60 ± 2.00	105.80 ± 7.00	99.97 ± 0.48	93.40 ± 1.50
Methylparaben	102.62 ± 2.50	94.10 ± 2.40	104.40 ± 0.70	100.69 ± 1.10	100.10 ± 0.34	94.23 ± 0.03
Propylparaben	104.00 ± 1.29	100.50 ± 1.59	99.98 ± 10.40	102.59 ± 10.3	100.50 ± 0.60	95.32 ± 3.90
Titanium dioxide	104.00 ± 0.80	103.20 ± 2.20	106.40 ± 2.57	103.69 ± 6.30	99.23 ± 0.76	99.19 ± 1.97
Transcutol^®^	103.53 ± 1.90	103.00 ± 0.86	103.40 ± 6.28	105.55 ± 2.00	100.65 ± 0.98	99.16 ± 4.40
Benzyl alcohol	109.90 ± 1.04	109.10 ± 7.45	108.63 ± 6.63	106.06 ± 8.84	108.18 ± 1.23	105.80 ± 1.40
Tween^®^ 20	106.60 ± 7.60	110.21 ± 2.53	102.86 ± 6.41	106.94 ± 6.91	104.58 ± 7.53	104.80 ± 2.60

Regarding drug–drug compatibility, DSC curves of LDH, NIF, and BMV individually showed respective endothermic peaks at 77.15, 169.94, and 194.50 °C representing the corresponding melting points (Figure 1(a)). The binary mixtures of LDH-NIF and LDH-BMV exhibited one peak at 73.49 and 75.30 °C, respectively, corresponding to that of LDH, while those of NIF and BMV disappeared. These results can be referred to the dissolution of the crystalline NIF and BMV in the molten LDH and complete conversion into the amorphous state during DSC run (Liu et al. 2013). The ternary mixture of LDH, NIF, and BMV exhibited three *T*_peak_ values at 73.80, 167.20, and 180 °C, respectively (Figure 1(b)). These small shifts in *T*_peak_ values of LDH and NIF could be attributed to the reduced purity of each component in the mixture and does not necessarily indicate incompatibility (Botha & Lötter 1990). While the larger shift in *T*_peak_ of BMV can be referred to the partial dissolution of this drug in the molten mass of the other two drugs.

Thermograms of the binary mixtures of each of LDH, NIF, and BMV with the different solid excipients are illustrated in Figure 1(c–e), respectively. In case of their binary systems with each of titanium dioxide and CP940, the characteristic drug’s peak appeared either without shifting or with minor shifting. On the other hand, peaks of NIF and BMV were absent in their binary mixtures with each of P407, methylparaben, and propylparaben. Endothermic peak around 56 °C in P407 DSC curve has been reported (Bei et al. 2010). Endothermic peaks of methylparaben and propylparaben have been found to be around 124 and 100 °C, respectively (Lira et al. 2007). Thus, disappearance of NIF and BMV endothermic peaks may be attributed to the dissolution in the molten excipient (P407, methylparaben, or propylparaben) of much lower melting point (Liu et al. 2013). LDH peak was shifted to ≈50 °C in its binary mixtures with methylparaben and propylparaben possibly due to the diminished purity of each component in the mixture (Botha & Lötter 1990). Disappearance or shift of the drugs peaks did not confirm the incompatibility particularly there were no new peaks.

### Fourier transform infrared spectroscopy (FT-IR)

FT-IR spectra of different blends of the three drugs and each of them with the excipients retained all characteristic bands of the drugs without appearance of new bands suggesting that these compounds are compatible (data are not shown).

### Isothermal stress testing (IST)

At the end of the IST (4 weeks), the percentage drug remained in test samples were compared to those of the corresponding control samples (Table 1(b)). As well, FT-IR spectra of stressed test samples were compared to those of corresponding control samples and only spectra that encountered changes are illustrated in Figure 1(f–h).

Both individual drugs and drug–drug mixtures exhibited insignificant changes in percentage drug remained after being subjected to IST. These samples did not show any changes in the organoleptic parameters (color and odor) throughout the storage period. In accordance, FT-IR spectra of stressed drugs and drug–drug binary mixtures did not show a shift or a disappearance of absorption bands when compared to those of control samples. Also, no appearance of new bands was noted. This furtherly indicated the compatibility of the three drugs.

Similarly, binary mixtures of LDH, NIF or BMV with each of CP940, titanium dioxide, Transcutol^®^, benzyl alcohol, and Tween^®^ 20 exhibited insignificant changes in percentage drug remained after being subjected to IST (Table 1(b)). There was no alteration in the organoleptic parameters of the stressed binary mixtures of the drugs with the above-mentioned excipients throughout the storage period. Accordingly, FT-IR spectra of stressed samples showed characteristic absorption bands comparable with those of control samples. These data strongly indicated the compatibility of the three drugs with CP940, titanium dioxide, Transcutol^®^, benzyl alcohol, and Tween^®^ 20.

In spite of the insignificantly different LDH content in the control and test samples, incompatibility of LDH with each of P407, methylparaben, and propylparaben can be suggested by characteristic odor and viscous liquid observed in case of stressed binary systems with each of these excipient possibly due to the acid degradation of the excipients in the acidic microenvironment provided by acid content in LDH without affecting LDH content in the binary mixtures (Rowe et al. 2009). In agreement, FT-IR spectrum of stressed LDH-P407 mixture showed a new peak at 1741 cm^−1^ (Figure 1(f)). In comparison with the control sample, the peak at 2516–2483 cm^−1^ disappeared at FT-IR spectra of LDH-methylparaben and LDH-propylparaben subjected to isothermal stress (Figure 1(g,h), respectively). This may indicate the incompatibility between LDH and these excipients. It can be said that NIF was compatible with these excipients as clarified by insignificantly different drug content of control and stressed test mixtures (Table 1(b)) as well as unchanged organoleptic properties on the storage. In addition, FT-IR spectra of these mixtures after storage at stress condition did not show new bands, band shift or absence of bands existing in spectra of control mixtures. BMV formed a milky liquid with P407 only on the storage. Yet, there was insignificant difference in BMV content in test and control mixtures with P407. Moreover, there were no detectable differences in FT-IR spectra of control and test mixtures. This may reflect only a physical incompatibility between BMV and P407.

Collectively, the results of DSC, FT-IR of unstressed systems, and IST clearly reflected the compatibility of the three drugs and each drug with the tested excipients except with LDH and each of methylparaben, propylparaben, and P407 as well as BMV with P407.

## Photostability study

**Figure 2.**
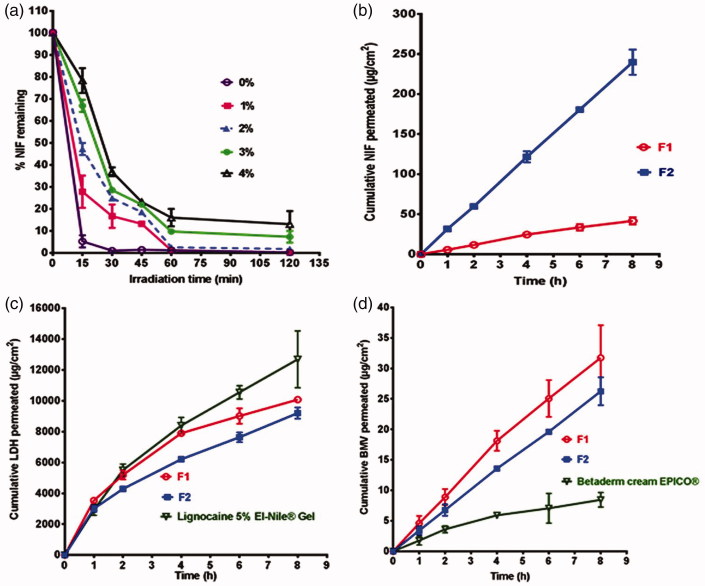
(a) Effects of irradiation on NIF photostability in gel formulations containing different concentrations of titanium dioxide. (b–d) *In vitro* release of (b) NIF, (c) LDH, and (d) BMV in phosphate buffer pH 7.4.

According to Figure 2(a), there was an improvement in NIF photostability on the increase in titanium dioxide concentration. Thus, titanium dioxide at a concentration of 4% w/w was used as photostabilizer by reducing photocatalysis activity (Lodén et al. 2005).

## *In vitro* evaluation of the topical formulations

### Viscosity, pH, and drug content

The formulations possessed acceptable viscosity ranges (Garg et al. 2015), pH values suitable for rectal application with minimal risk of tissue irritation (El-Leithy et al. 2010) and drugs content ranges within the pharmacopoeial limit of 90–110% of the label claim (B.P. 2010) (Table 2(a)).

**Table 2.** *In vitro* evaluation of the prepared combination gels (F1 and F2) and the market products (a) Viscosity, pH, drug content (%), and steady-state flux (*J*_ss_). (b) Kinetic modeling of release data. (c) Stability study data of the optimized combination gel (F2) after storage for three months at different temperatures.

(a)								
Formula	Viscosity (mPa.s)	pH Mean ± SD	Drug content (%), Mean ± SD			*J*_ss_ (µg/cm^2^.h), Mean ± SD		
			NIF	LDH	BMV	NIF	LDH	BMV
F1	1858 ± 22	5.95 ± 0.03	102.9 ± 2.6	97.8 ± 1.8	99.21 ± 3.1	5.31 ± 0.22	1161 ± 103	4.00 ± 0.20
F2	2043 ± 34	5.88 ± 0.02	102.6 ± 1.12	99.9 ± 1.8	104.3 ± 1.3	29.97 ± 0.5	1045 ± 71	3.27 ± 0.079
Lignocaine^®^ 5% gel	1479 ± 26	6.05 ± 0.008	–	101.5 ± 3.1	–	–	1518 ± 99	–
Betaderm^®^ cream	2237 ± 34	4.76 ± 0.04	–	–	99.27 ± 1.0	–	–	1.03 ± 0.10

(b)								
		The correlation coefficient (*r*^2^)				Korsmeyer		Main transport mechanism
Drug	Formula	Zero-order	First order	Higuchi model	Release order	*r*^2^	*n*	
NIF	F1	0.9712	0.8798	0.9171	Zero order	0.9850	0.97	Non-Fickian
	F2	0.9952	0.9299	0.9119	Zero order	0.9964	0.98	Non-Fickian
LDH	F1	0.8873	0.8705	0.9907	Diffusion	0.9838	0.51	Non-Fickian
	F2	0.9314	0.9327	0.9944	Diffusion	0.9917	0.53	Non-Fickian
	Lignocaine^®^ 5% gel	0.9363	0.8533	0.9622	Diffusion	0.9656	0.68	Non-Fickian
BMV	F1	0.9589	0.8752	0.9032	Zero order	0.9640	0.95	Non-Fickian
	F2	0.9907	0.9092	0.9120	Zero order	0.9846	0.99	Non-Fickian
	Betaderm^®^ cream	0.8617	0.7317	0.8864	Diffusion	0.8573	0.75	Non-Fickian

(c)									
Storage Temp.	NIF			LDH			BMV		
	*t*_90_	*t*_50_	% Drug remained	*t*_90_	*t*_50_	% Drug remained	*t*_90_	*t*_50_	% Drug remained
(°C)	(days)	(days)	Mean ± SD	(days)	(days)	Mean ± SD	(days)	(days)	Mean ± SD
8 ± 1	910	3920	101.77 ± 1.16	1141	5084	101.42 ± 2.77	845	3664	100.77 ± 0.86
25 ± 1	691	2917	100.66 ± 2.96	806	3546	100.61 ± 4.42	78	320	89.77 ± 2.02
40 ± 1	434	1821	100.29 ± 2.42	331	1371	101.26 ± 4.27	5	66	43.37 ± 4.33

### *In vitro* release study

The prepared combination gel containing 10% Transcutol^®^ (F2) showed significantly (*p* < 0.05) higher release of NIF compared to that observed with the formula without Transcutol^®^ (F1) (Figure 2(b)). In agreement, *J*_ss_ of NIF from F2 was 5.64 folds higher than that of F1 gel formulation (Table 2(a)). Enhancement of NIF transport rate could be related to the solubilizing action and penetration enhancement of Transcutol^®^ (Santis et al. 2013). On the other hand, *in vitro* release of both LDH and BMV insignificantly affected by the incorporation of Transcutol^®^ (Figure 2(c,d), respectively). Accordingly, there was insignificant difference between *J*_ss_ values of these drugs in the two gel formulations (Table 2(a)). *In vitro* release of LDH from the prepared gels was comparable with that of the market gel (Figure 2(c)). In case of BMV, the drug permeation from these gels was superior to that seen with the market product (Figure 2(d)). The values of *J*_ss_ for these drugs reflected such behavior (Table 2(a)). The results may represent F2 combination gel with 10% w/w Transcutol^®^ as an optimized formula to be furtherly investigated regarding stability and efficacy in treatment of anal fissure.

### Release kinetics

The data of kinetic analysis are represented in Table 2(b). The drugs release mechanism followed either zero kinetics or diffusion transport. To verify it, the release data were analyzed *via* Korsmeyer–Peppas equation. Non-Fickian diffusion (0.5 > *n* > 1) predominated suggesting that a combination of diffusion and polymer chain relaxation contributed to the overall release of the drugs (Ritger & Peppas 1987).

### Stability study

In comparison with the initial measurements, there were insignificant changes in pH (5.53 ± 0.03 to 5.56 ± 0.016) and viscosity (2126.30 ± 13.07 to 2191.01 ± 22.64) of the optimized gel (F2) during the whole storage period (3 months).

Similarly, percentage NIF and LDH content remained within the pharmacopoeial limit (90–110%) up to the end of storage period at the different storage temperatures (Table 2(c)). In contrary, percentage BMV remained significantly (*p* < 0.05) decreased at 25 ± 1 and 40 ± 1 °C relative to that determined at 8 ± 1 °C. Generally, there was a sharp decline in *t*_90_ and *t*_50_ of the drugs on the elevation of storage temperature particularly with BMV. Accordingly, the highest *t*_90_ and *t*_50_ values of the three drugs were obtained on the storage at refrigerator (8 ± 1 °C). Moreover, the optimized gel preparation stored at the three storage temperatures did not encounter bacterial or fungal growth. Collectively, the results revealed that the recommended storage temperature was 8 ± 1 °C.

## Clinical study

A prospective randomized controlled study has been conducted on 37 patients with AAF and 34 patients with CAF. Baseline characteristics of the study population are listed in Table 3(a). Prevalence of fissure healing, pain, bleeding, discharge, and itching among patients with AAF and CAF at first, third, and sixth weeks post-treatment are demonstrated in Table 3(b).

**Table 3.** Clinical study (a) Baseline characteristics of the study population. (b) Prevalence of fissure healing, pain, bleeding, discharge, and itching among patients with AAF and CAF at first, third, and sixth week post-treatment.

(a)					
	Total (*n* = 71)	AAF (*n* = 37)		CAF (*n* = 34)	
		Group A	Group B	Group C	Group D
		(*n* = 20)	(*n* = 17)	(*n* = 18)	(*n* = 16)
Age (years)	33.50 ± 8.80	37.40 ± 10.10	31.50 ± 6.10	33.70 ± 9.60	30.60 ± 7.30
Gender					
Male *n* (%)	22 (31.0%)	7 (35.0%)	5 (29.4%)	6 (33.3%)	4 (25.0%)
Female *n* (%)	49 (69.0%)	13 (65.0%)	12 (70.6%)	12 (66.7%)	12 (75.0%)
Duration of symptoms (weeks)	8 (1–55)	4 (1–8)	4 (1–8)	18 (11–55)	25.5 (12–55)
Pain scale	90 (50–100)	90 (80–100)	90 (80–100)	80 (50–100)	80 (60–100)
Patients have bleeding (%)	15 (21.1%)	1 (5.0%)	2 (11.7%)	6 (33.30)	6 (37.0%)
Bleeding, discharge, and itching	8 (6–9)	9 (6–9)	9 (6–9)	6 (6–9)	6 (6–9)

(b)								
No of weeks Symptoms	AAF				CAF			
	Total	Group A	Group B		Total	Group C	Group D	
	(*n* = 37)	(*n* = 20)	(*n* = 17)	*p* Value	(*n* = 34)	(*n* = 18)	(*n* = 16)	*p* Value
First week								
Healing *n* (%)								
Complete	0 (0.0%)	0 (0.0%)	0 (0.0%)	0.1^a^	0 (0.0%)	0 (0.0%)	0 (0.0%)	0.004^a^
Partial	32 (86.5%)	19 (95.0%)	13 (76.5%)		28 (82.4%)	18 (100.0%)	10 (62.5%)	
No	5 (13.5%)	1 (5.0%)	4 (23.5%)		6 (17.6%)	0 (0.0%)	6 (37.5%)	
pain	50 (40–70)	50 (40–70)	60 (40–70)	0.115^b^	60 (30–90)	50 (30–70)	60 (30–90)	0.223^b^
Bleeding discharge/itching	6 (3–8)	4.5 (3–8)	6 (3–6)	0.728^b^	6 (3–6)	6 (3–6)	6 (3–6)	0.436^b^
Third week								
Healing *n* (%)								
Complete	27 (73.0%)	18 (90.0%)	9 (52.9%)	0.024^a^	24 (70.6%)	15 (83.3%)	9 (56.3%)	0.015^a^
Partial	6 (16.2%)	2 (10.0%)	4 (23.5%)		4 (11.8%)	3 (16.7%)	1 (6.3%)	
No	4 (10.8%)	0 (0.0%)	4 (23.5%)		6 (17.6%)	0 (0.0%)	6 (37.5%)	
pain	20 (10–80)	20 (10–50)	40 (10–80)	0.007^b^	20 (10–80)	20 (10–60)	40 (10–80)	0.053^b^
Bleeding discharge/itching	3 (0–7)	3 (0–7)	3 (0–6)	0.249^b^	3 (0–6)	3 (0–6)	4.5 (3–6)	0.031^b^
Sixth week								
Healing *n* (%)								
Complete	28 (75.7%)	19 (95.0%)	9 (52.9%)	0.01^a^	24 (70.6%)	15 (83.3%)	9 (56.3%)	0.015^a^
Partial	4 (10.8%)	0 (0.0%)	4 (23.5%)		4 (11.8%)	3 (16.7%)	1 (6.3%)	
No	5 (13.5%)	1 (5.0%)	4 (23.5%)		6 (17.6%)	0 (0.0%)	6 (37.5%)	
pain	20 (10–70)	10 (10–50)	20 (10–70)	0.001^b^	15 (10–90)	10 (10–50)	20 (10–90)	0.023^b^
Bleeding discharge/itching	1 (0–6)	0 (0–6)	2 (0–6)	0.004^b^	1.5 (0–9)	0 (0–6)	3 (0–9)	0.042^b^

a Chi-square test.

b Mann–Whitney *U* test.

Group A; with acute anal fissure (AAF) treated with the optimized combination gel (F2).

Group B; with acute anal fissure (AAF) treated with the market topical preparations.

Group C; with chronic anal fissure (CAF) treated with the optimized combination gel (F2).

Group D; with chronic anal fissure (CAF) treated with the market topical preparations.

There was no significant difference in the baseline characteristics of the participated patients. The mean age of patients was 33.5 ± 8.8 years, with 22 males and 49 females. Regarding the symptomatology, pain, discharge, and itching were more frequent than rectal bleeding (Table 3(a)).

**Figure 3.**
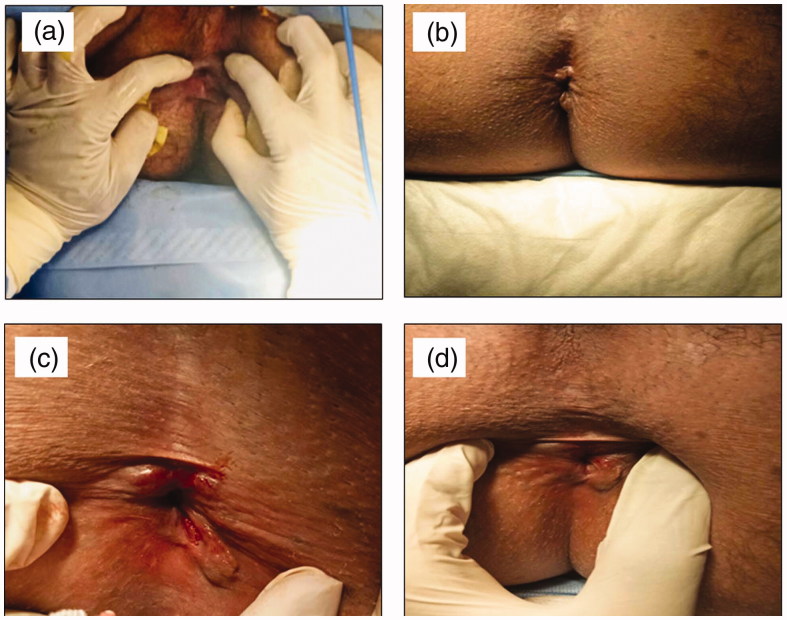
Representative photographs of patients suffering from either AAF (a) before treatment (baseline), (b) after treatment for six weeks with the optimized combination gel (F2) or CAF, (c) before treatment (baseline), and (d) after treatment for six weeks with the optimized combination gel (F2).

There was a significant difference in AAF healing between the two groups (A&B) at third (*p* = .024) and sixth (*p* = 0.01) weeks post-treatment. There was a significant difference in CAF healing between the two groups (C&D) at first (*p* = 0.004), third (*p* = 0.015), and sixth (*p* = 0.015) weeks post-treatment. Complete healing of AAF occurred in 95.0% of patients in group A against 52.9% in group B and 83.3% of patients in group C against 56.3% in group D by the end of the sixth week (Table 3(b)). Three patients in group D were unsatisfied and subjected to anal dilation and sphincterotomy. Hence, it can be concluded that the optimized gel (F2) provided more effective healing of AAF (group A) and CAF (group C) than the three topical market preparations (groups B and D, respectively). The effective healing of patients suffering from either AAF or CAF after treatment with the optimized combination gel (F2) for six weeks is illustrated in Figure 3.

There was a higher severity of pain among AAF patients (median =90) in comparison with CAF patients (median 80) (Table 3(a)). Although there were equal medians of pain baseline scale in case of group A and B patients on one side and group C and D patients on the other side (Table 3(a)), the severity of pain reflected by the median that became lower among groups A and C patients treated with the optimized gel in comparison with groups B and D treated with the market products (Table 3(b)). The median of pain score dropped from 90 (baseline value) to 10 and 20 in groups A and B, respectively, at the sixth-week post-treatment with statistically significant difference between the two groups (*p* = 0.001). In addition, it dropped from 80 (baseline value) to 10 and 20 in groups C and D, respectively, at the sixth-week post-treatment with statistically significant difference between the two groups (*p* = 0.023) (Table 3(b)). These results indicated that the optimized gel was more effective to relieve pain in patients with AAF (group A) or CAF (group C) than the three topical market preparations (groups B and D).

Regarding the basic characteristics of the population, there was a higher prevalence of rectal bleeding among patients with CAF in comparison with those with AAF. Although there was a higher severity of anal discharge and itching among AAF patients in comparison with CAF patients, equal medians of their scales were observed between groups A and B patients on one side and between groups C and D patients on the other side. In comparison with groups B and D treated with market products, the severity of rectal bleeding, anal discharge, and itching became lower among groups A and C patients treated with the optimized combination gel formulation. The median of rectal bleeding, anal discharge, and itching scores dropped from 9 (basic value) to 0 and 2 in groups A and B, respectively, at the sixth-week post-treatment with statistically significant difference between the two groups (*p* = 0.004). Similarly, it dropped from 6 to 0 and 3 in groups C and D, respectively, at the sixth-week post-treatment with statistically significant difference between the two groups (*p* = 0.042) (Table 3(b)).

No patients in groups treated with optimized gel reported side effects (groups A and C). While mild headache was experienced by 24.2% of patients treated with market formulations (groups B and D). Perianal itching was another side effect, which was reported by 9.1% of the cases in market formulations treated groups. Both headache and perianal itching were reported by 6.1% of patients in market formulations treated groups

There was no significant difference in healing of anal fissure as well as relief of pain, rectal bleeding, anal discharge, and itching following treatment with the optimized formula among AAF patients (group A) in comparison with CAF patients (group C).

## Conclusions

The compatibility among the three drugs and between the drugs and tested excipients was confirmed except with LDH and each of P407, methylparaben, and propylparaben as well as BMV and P407. The gels containing compatible excipients showed acceptable viscosity and pH ranges as well as pharmacopoeial complying drugs content limits. The optimized combination gel incorporating 10% Transcutol^®^ (F2) exhibited significantly (*p* < 0.05) enhanced NIF release. Compared to the tested single drug market products of glyceryl trinitrate, LDH, and BMV, optimized formula provided more potentiated efficacy in the treatment of anal fissure either acute or chronic as clarified by significantly increased healing% and significantly reduced pain, bleeding, anal discharge, and itching. In addition, no side effects were reported by patients treated with the optimized combination gel in contrast to those treated with the market products.

## References

[CIT0001] Agrawal V, Kaushal G, Gupta R (2013). Randomized controlled pilot trial of nifedipine as oral therapy vs. topical application in the treatment of fissure-in-ano. Am J Surg 206:748–51. 24035211 10.1016/j.amjsurg.2013.05.003

[CIT0002] Aman W, Thoma K (2003). ICH guideline for photostability testing: aspects and directions for use. Pharmazie 58:877–80. 14703965

[CIT0003] Arafa MG, Ayoub BM (2017). DOE Optimization of nano-based carrier of pregabalin as hydrogel: new therapeutic; chemometric approaches for controlled drug delivery systems. Sci Rep 7:41503. 28134262 10.1038/srep41503 PMC5278417

[CIT0004] B.P. (British Pharmacopoeia Commission). (2010). British pharmacopeia. Vol. III, 6th ed London: The Council of Europe, The Stationary Office, 3155–7.

[CIT0005] Beaty JS, Shashidharan M (2016). Anal fissure. Clin Colon Rectal Surg 29:30–7. 26929749 10.1055/s-0035-1570390 PMC4755763

[CIT0006] Bei D, Zhang T, Murowchick JB, Youan BB (2010). Formulation of dacarbazine-loaded cubosomes. Part III. Physicochemical characterization. AAPS Pharm 11:1243–9. 10.1208/s12249-010-9496-7 PMC2974159 20694534

[CIT0007] Botha SA, Lötter AP (1990). Compatibility study between naproxen and tablet excipients using differential scanning calorimetry. Drug Dev Ind Pharm 16:673–83.

[CIT0008] Bulus H, Varol N, Tas A, Coskun A (2013). Comparison of topical isosorbide mononitrate, topical diltiazem, and their combination in the treatment of chronic anal fissure. Asian J Surg 36:165–9. 24054756 10.1016/j.asjsur.2013.01.010

[CIT0009] Byrne J, Wyraz A, Velasco-Torrijos T, Reinhardt R (2017). Formulation factors affecting the isomerization rate of betamethasone-17-valerate in a developmental hydrophilic cream–a HPLC and microscopy based stability study. Pharm Dev Technol 22:537–44. 26895450 10.3109/10837450.2016.1143003

[CIT0010] Collins SL, Moore RA, Mcquay HJ (1997). The visual analogue pain intensity scale: what is moderate pain in millimetres? Pain 72:95–7. 9272792 10.1016/s0304-3959(97)00005-5

[CIT0011] Das B, Nayak AK, Nanda U (2013). Topical gels of lidocaine HCl using cashew gum and Carbopol 940: preparation and *in vitro* skin permeation. Int J Biol Macromol 62:514–7. 24099938 10.1016/j.ijbiomac.2013.09.049

[CIT0012] Denyer S, Hodges N (2004). Sterilization procedures and sterility assurance In: Denyer SP, Hodges NA, Gorman SP, editors. Hugo & russell’s-pharmaceutical microbiology. 7th ed Hoboken (NJ): Blackwell Science, 346–75.

[CIT0013] Eggleston ST, Lush LW (1996). Understanding allergic reactions to local anesthetics. Ann Pharmacother 30:851–7. 8826570 10.1177/106002809603000724

[CIT0014] El-Leithy ES, Shaker DS, Ghorab MK, Abdel-Rashid RS (2010). Evaluation of mucoadhesive hydrogels loaded with diclofenac sodium-chitosan microspheres for rectal administration. AAPS Pharm 11:1695–702. 10.1208/s12249-010-9544-3 PMC3011065 21108027

[CIT0015] Garg T, Rath G, Goyal AK (2015). Comprehensive review on additives of topical dosage forms for drug delivery. Drug Deliv 22:969–87. 24456019 10.3109/10717544.2013.879355

[CIT0016] Hall G, Kann BR (2016). Anal fissure. Anorectal disease. Berlin, Germany: Springer, 95–126.

[CIT0017] Haq Z, Rahman M, Chowdhury R, et al. (2005). Chemical sphincterotomy-first line of treatment for chronic anal fissure. Mymensingh Med J 14:88–90. 15695964

[CIT0018] Havlickova B (2010). Topical corticosteroid therapy in proctology indications. Aliment Pharm Ther 31:19–32.

[CIT0019] Higuchi T (1963). Mechanism of sustained-action medication. Theoretical analysis of rate of release of solid drugs dispersed in solid matrices. J Pharm Sci 52:1145–9. 14088963 10.1002/jps.2600521210

[CIT0020] Hooda A, Nanda A, Jain M, et al. (2012). Optimization and evaluation of gastroretentive ranitidine HCl microspheres by using design expert software. Int J Biol Macromol 51:691–700. 22903013 10.1016/j.ijbiomac.2012.07.030

[CIT0021] Khunt DM, Mishra AD, Shah DR (2012). Formulation design & development of piroxicam emulgel. Int J Pharmtech Res 4:1332–44.

[CIT0022] Late S, Banga A (2008). Thermal and non-thermal methods to evaluate compatibility of granisetron hydrochloride with tablet excipients. Pharmazie 63:453–8. 18604989

[CIT0023] Lira A, Araújo A, Basílio I, et al. (2007). Compatibility studies of lapachol with pharmaceutical excipients for the development of topical formulations. Thermochim Acta 457:1–6.

[CIT0024] Liu J, Cao F, Zhang C, Ping Q (2013). Use of polymer combinations in the preparation of solid dispersions of a thermally unstable drug by hot-melt extrusion. Acta Pharm Sin B 3:263–72.

[CIT0025] Lodén M, Åkerström U, Lindahl K, Berne B (2005). Novel method for studying photolability of topical formulations: a case study of titanium dioxide stabilization of ketoprofen. J Pharm Sci 94:781–7. 15729703 10.1002/jps.20295

[CIT0026] Maafi W, Maafi M (2013). Modelling nifedipine photodegradation, photostability and actinometric properties. Int J Pharm 456:153–64. 23954300 10.1016/j.ijpharm.2013.07.075

[CIT0027] Martin AN, Bustamante P, Chun AHC (1993). Physical pharmacy: physical chemical principles in the pharmaceutical sciences. 4th ed Philadelphia: Lea & Febiger.

[CIT0028] Moffat AC, Osselton MD, Widdop B, Watts J (2011). Clarke’s analysis of drugs and poisons. 4th ed London: Pharmaceutical Press.

[CIT0029] Nagajyothi M, Pramod K, Bijin E, et al. (2015). Accelerated stability studies of atorvastatin loaded nanoemulsion gel. Asian J Pharm Technol 5:188–91.

[CIT0030] Perrotti A, Bartone N, Stefano MD, et al. (2000). Topical Nifedipine^®^ for conservative treatment of acute haemorrhoidal thrombosis. Colorectal Dis 2:18–21. 23577929 10.1046/j.1463-1318.2000.00130.x

[CIT0031] Perrotti P, Bove A, Antropoli C, et al. (2002). Topical nifedipine with lidocaine ointment vs. active control for treatment of chronic anal fissure: results of a prospective, randomized, double-blind study. Dis Colon Rectum 45:1468–75. 12432293 10.1007/s10350-004-6452-1

[CIT0032] Ritger PL, Peppas NA (1987). A simple equation for description of solute release I. Fickian and non-Fickian release from non-swellable devices in the form of slabs, spheres, cylinders or discs. J Control Release 5:23–36. 25356469

[CIT0033] Rowe RC, Sheskey PJ, Quinn ME (2009). Handbook of pharmaceutical excipients. 6th ed Washington (DC): American Pharmaceutical Association.

[CIT0034] Santis AK, De Freitas ZMF, Ricci-Junior E, et al. (2013). Nifedipine in semi-solid formulations for topical use in peripheral vascular disease: preparation, characterization, and permeation assay. Drug Dev Ind Pharm 39:1098–106. 22901029 10.3109/03639045.2012.711833

[CIT0035] Shenfield GM (1982). Fixed combination drug therapy. Drugs 23:462–80. 7049658 10.2165/00003495-198223060-00003

[CIT0036] Srivastava A, Wadhwa S, Ridhurkar D, Mishra B (2005). Oral sustained delivery of atenolol from floating matrix tablets-formulation and in vitro evaluation. Drug Dev Ind Pharm 31:367–74. 16093202 10.1081/ddc-54313

[CIT0037] Torsiello M, Kopacki M (2000). Transdermal nifedipine for wound healing: case reports. Int J Pharm Compd 4:356–8. 23981700

[CIT0038] Tsunoda A, Kashiwagura Y, Hirose KI, et al. (2012). Quality of life in patients with chronic anal fissure after topical treatment with diltiazem. World J Gastrointest Surg 4:251–5. 23494072 10.4240/wjgs.v4.i11.251 PMC3596506

[CIT0039] Verma RK, Garg S (2005). Selection of excipients for extended release formulations of glipizide through drug-excipient compatibility testing. J Pharm Biomed Anal 38:633–44. 15967291 10.1016/j.jpba.2005.02.026

[CIT0040] Yang H, Zhu S, Pan N (2004). Studying the mechanisms of titanium dioxide as ultraviolet-blocking additive for films and fabrics by an improved scheme. J Appl Polym Sci 92:3201–10.

[CIT0041] Zimmermann J, Schlegelmilch R, Mazur D, et al. (2007). Proof of systemic safety of a lidocaine ointment in the treatment of patients with anorectal pain. Arzneimittelforschung 57:12–19. 17341004 10.1055/s-0031-1296580

